# Combination Therapy With Anti-PD-1 or PD-1 Antibody Alone in Asian Pediatric Patients With Relapsed or Refractory Cancer

**DOI:** 10.3389/fimmu.2021.647733

**Published:** 2021-07-06

**Authors:** Yi Que, Juan Wang, Jia Zhu, Na Li, Junting Huang, Suying Lu, Feifei Sun, Lian Zhang, Zijun Zhen, Li Zhang, Ruiqing Cai, Haixia Guo, Xiaofei Sun, Yizhuo Zhang

**Affiliations:** ^1^ Sun Yat-sen University Cancer Center, State Key Laboratory of Oncology in South China, Collaborative Innovation Center for Cancer Medicine, Guangzhou, China; ^2^ Department of Pediatric Oncology, Sun Yat-sen University Cancer Center, Guangzhou, China; ^3^ Department of Pediatrics, Nanfang Hospital, Southern Medical University, Guangzhou, China

**Keywords:** PD-1 antibody, pediatric cancer, monotherapy, combination, PD-L1

## Abstract

There is limited experience of PD-1 antibody combined with other therapies in children. We aimed to explore the antitumor activity and safety of PD-1 antibody monotherapy or combination with other regimens in relapsed or refractory pediatric cancer. This is a retrospective-case study conducted in two Chinese expert centers. The primary objective of this study was to describe the overall response rate (ORR) and disease control rate (DCR). Secondary objectives included characterizing toxicities. Of the 22 pediatric patients with cancer who received PD-1 inhibitors, the median follow-up for all patients after the commencement of PD-1 therapy with or without other regimens was 12.3 months (0 - 43 months). PD-1 antibody monotherapy demonstrated antitumor activity in a population of pediatric patients with Hodgkin lymphoma (HL), with an objective response rate (ORR) and disease control rate (DCR) of 83.3% (3CR and 2PR) and 100%, respectively. However, no objective response was observed in patients with melanoma or Burkitt lymphoma evaluated in this study. We reviewed responses for patients with chemotherapy, decitabine or everolimus combination therapies with PD-1 antibodies, and found that PD-1 antibody combined with decitabine showed potential efficacy in pediatric patients with advanced embryonal rhabdomyosarcoma and lymphoepitheliomatoid-like carcinoma. There were no severe treatment-related adverse events (TRAEs) directly attributed to PD-1 antibody monotherapy in Asian pediatric patients with lower incidence of hematologic toxicity and nonhematologic toxicity. The Grade ≥3 TRAEs were attributed to the combination chemotherapy.

## Introduction

For pediatric patients with cancer, the standard treatment includes surgery, nonspecific cytotoxic chemotherapy and radiotherapy (1, 2). However, the prognosis for recurrent/progressive solid tumors in children remains unfavorable, with a 10-year OS and PFS of 24.5% and 18.4%, respectively (3). Thus, novel development approaches for pediatric cancers are urgently needed (4, 5). In particular, immune checkpoint inhibitors targeting PD-1 have achieved great success in adult patients with cancer (6–8). Currently, there is limited experience regarding the safety and efficacy of PD-1 antibodies in pediatric patients especially in Asian populations. In the KEYNOTE-051 study, 12 subjects of South Korea were included but all of them received PD-1 antibody monotherapy (9), which supports the exploration of the potential of combination therapy with anti-PD-1 or PD-1 antibody alone in relapsed or refractory pediatric cancer from a real-world perspective.

To date, inhibitors of pembrolizumab and nivolumab have been respectively explored prospectively in clinical trials (9, 10) involving pediatric patients (KEYNOTE-051 & ADVL1412). The results indicated that pembrolizumab and nivolumab are safe and tolerated in pediatric patients while the clinical activity is observed in lymphoma especially in HL. However, there is no significant single-agent activity in the common pediatric solid tumors, which provides a basis for combinatorial therapies for PD-1 antibodies.

Toripalimab and sintilimab are two recombinant and humanized PD-1 monoclonal antibodies that were developed in China in 2018 (11, 12). Toripalimab has received conditional approval in China for the treatment of unresectable or metastatic melanoma that has failed previous systemic therapy (13) and sintilimab has been approved for the treatment of classical HL in patients who have relapsed or are refractory after ≥2 lines of systemic chemotherapy (14). However, there are no reports of their efficacy or safety for treatment in pediatric patients with cancer.

## The Efficacy and Safety of PD-1 Antibody Alone or Combination in Pediatric Patients With Cancer

To the best of our knowledge, no evidence of PD-1 antibody combination treatment has been previously reported in Asian pediatric patients. Recognizing the scarcity of data, we aimed to investigate the safety and antitumor activity of combination with anti-PD-1 or PD-1 antibody alone in pediatric cancer to capture real-world evidence.

We conducted an observational retrospective study which was performed at two academic medical centers (Sun Yat-sen University Cancer Center and Nanfang Hospital, Southern Medical University) evaluating pediatric patients who received a PD-1 inhibitor outside of a clinical trial between 2017 and 2020. The demographic, clinical and treatment data for each patient were obtained from retrospective electronic medical records by investigators. The inclusion criteria were as follows: (1) a diagnosis of pediatric malignant cancer confirmed by either radiographic features and/or pathology, and (2) initial diagnosis ≤ 18 years of age. Patients were excluded if they received a PD-1 antibody as part of a clinical trial. Our study was approved by the Ethic Committee of Sun Yat-sen University Cancer Center.

All the patients we included had measured disease at baseline. Responses were assessed according to RECIST 1.1 criteria (15, 16). For patients with lymphoma, the lugano classification was also used to evaluated response (17). The primary objective of this study was to describe the overall response rate (ORR) and disease control rate (DCR) on contrast enhanced CT/MRI or PET/CT. The disease control rate was defined as the proportion of patients who achieved a complete response (CR) or a partial response (PR) or stable disease (SD). The ORR was defined as the proportion of patients who achieved a CR or PR. Secondary objectives included characterizing toxicities according to the Common Terminology Criteria for Adverse Events (CTCAE) version 4.03. We included hematologic toxicity and nonhematologic toxicity, including hematological index, thyroid function, gastrointestinal toxicity, renal toxicity, hepatotoxicity, neurotoxicity, dermatologic toxicity. Serious adverse events were defined as any event that resulted in life threatening reactions or death, resulted in persistent disability, prolonged an existing inpatient hospitalization, or any other important medical event.

Twenty-two pediatric patients with cancer who received PD-1 inhibitors between 2017 and 2020 were enrolled. The median follow-up for all patients after commencement of anti-PD-1 treatment with other therapies was 12.3 months (0 - 43 months). No patients received previous checkpoint inhibitor therapy. The median age of all patients was 7.7 (1-15) years, and there was a male predominance (17/22). Most patients had at least 2 previous lines of therapy. The disease characteristics and treatment details for each patient in the study cohort are summarized in **Supplementary Table 1**.

**Figure 1:**
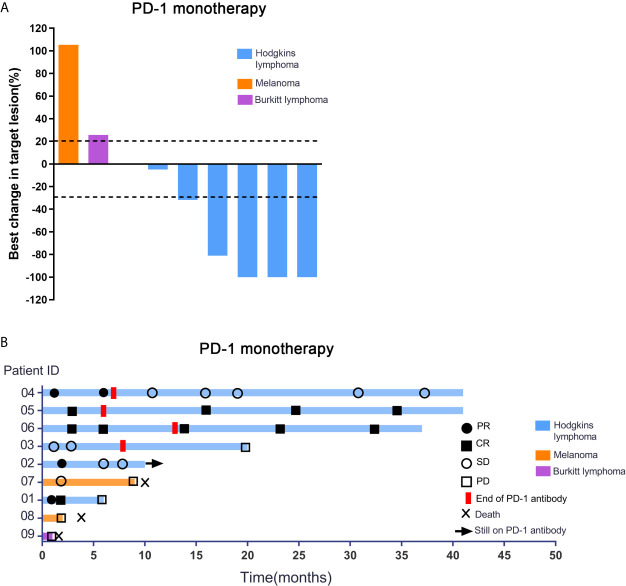
Response characteristics and changes in tumor burden in patients receiving PD-1 inhibitor alone. **(A)** Shown are best percentage changes from baseline in the sum of the longest diameter of target lesions in patients treated with PD-1 monotherapy with at least one post-baseline assessment. **(B)** Swimmer’s Plot of Time on Treatment for 9 evaluable Patients treated with PD-1 monotherapy. As shown in **Figure 1**, no objective response was observed in patients with melanoma or Burkitt lymphoma evaluated in this study. Stable disease was reported as the best response in patient 7. This patient had metastatic disease at the time of enrollment and had progressed despite two palliative surgical resections. After anti-PD-1 treatment, he achieved progression free survival for 9 months. Patient 8 progressed after treatment with 2 cycles of PD-1 antibody and the PFS was 62 days. Patient 9 achieved only 1 cycle of PD-1 antibody and progressed rapidly. Patient #7, 8 and 9 patients had eventually died due to tumor progression.

**Figure 2:**
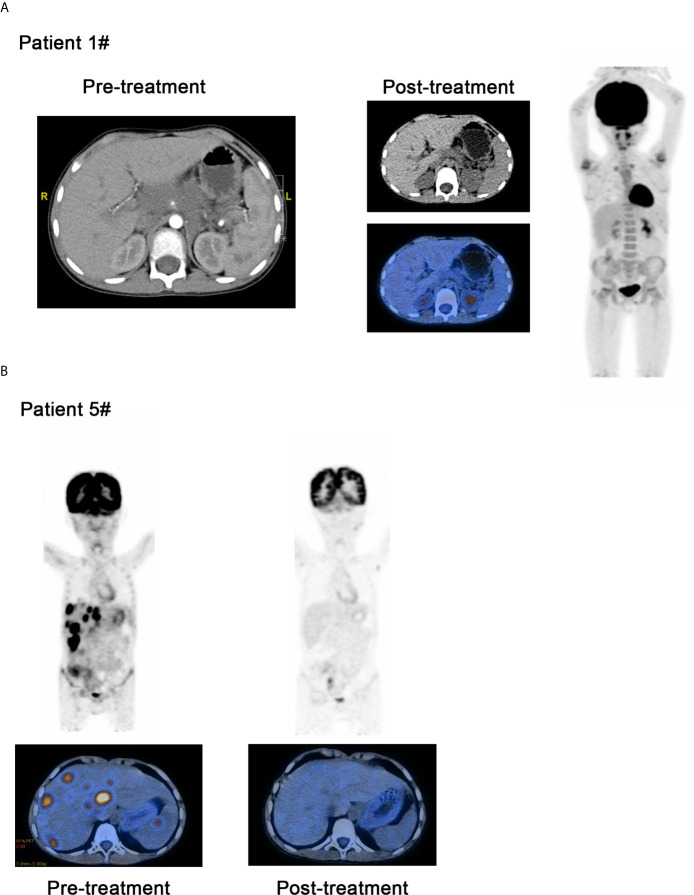
Comparison of curative effect of patients before and after treatment through image examination. **(A)** Patient 1 who was diagnosed as the Hodgkin lymphoma has achieved CR and sustained remission for 6 months after PD-1 inhibitor therapy. **(B)** Patient #5 was also achieved CR after 6 cycles of PD-1 inhibitor therapy and did not progressed by the data cutoff.

For the patients with Hodgkin lymphoma, the decrease in target lesion size was generally sustained over time in most cases. Five of the 6 patients with HL achieved an objective response (83.3%). The best overall responses were 3 CR and 2 PR. Three of 6 patients with HL had a metabolic CR, 2 of 6 had a metabolic PR and 1 had a metabolic SD by PET (17, 18). All patients showed a reduction from baseline in the size of their target lesion. All 6 patients with HL achieved disease control (100%). Among patients with HL who had an objective response to treatment, the mean percentage of tumor change was −69.6% (**Figure 1A**). The median time to objective response in the responders with Hodgkin lymphoma was 1 month (range 1-3 months). At the data cutoff, only 1 of the 5 responders with HL had ongoing responses and was still on treatment. In addition, 3 patients had achieved disease control and discontinued the anti-PD-1 treatment, and 2 patients with HL had progression-free survival events (disease progression) by the data cutoff (**Figure 1B**). For the 6 patients with HL, progression-free survival at 6 months was 100%, and it was 66.7% at 12 months. No deaths were reported in the population with HL. Patient #1 who was diagnosed with HL has achieved CR and sustained remission for 6 months after PD-1 antibody therapy (**Figure 2A**). Patient #5 was also achieved CR after 6 cycles of PD-1 antibody therapy and did not progress by the data cutoff (**Figure 2B**).

For patients treated with PD-1 inhibitor combination therapy, pathology with HL was not included. All 13 patients relapsed or were refractory and experienced failure of standard chemotherapy. Among the 13 patients who received combination therapy, 9 received PD-1 antibody combined with chemotherapy regimens, 3 received PD-1 antibody combined with decitabine and 1 received everolimus, an mTOR inhibitor. The results showed that only patients #10 (**Supplementary Figure 1**) and #19 achieved CR after receiving anti-PD-1 combination treatment. PR was noted as the best response for 4 of the 13 patients. Interestingly, 1 of the 3 of patients who received PD-1 antibody combined with decitabine achieved CR and another two other patients achieved PR. At the data cutoff, 10 of the 13 (76.9%) patients achieved disease control as the best objective response. The patients who had disease progression eventually died by the data cutoff. The best response changes in tumor burden and time on treatment in patients receiving combination therapies are shown in **Supplementary Figure 2**.

PD-1 antibody monotherapy was well tolerated overall in pediatric patients. There were no severe treatment-related adverse events that were directly attributed to PD-1 antibody monotherapy. Additionally, there were no treatment-related deaths. The full list of TRAEs in all the patients is shown in **Supplementary Table 2**.

## Discussion

Consistent with a previous report, pediatric patients with HL experienced clinical benefits from PD-1 antibodies (9). Of the 6 treated HL patients, 5 experienced an objective response (3 complete response, 2 partial response and 1 stable disease), which was 60% and 80% of the ORR and DCR in the KEYNOTE-051 trial, respectively (9). In adult HL patients, the objective response has been reported to be 87% of relapsed or refractory HL patients treated with nivolumab (19) and 69.0% of patients treated with pembrolizumab (20). The genetic mechanism might be the frequent alterations in chromosome 9p24.1, which leads to overexpression of PD-L1, resulting in HL being more susceptible to PD-1 blockade (21). There is no evidence for the single-agent activity of PD-1 inhibitors in pediatric melanoma. One explanation for this result might be the distinct biological diversity between pediatric and adult melanoma (22). Recent preliminary evidence has suggested that alterations in TERT (23–25) and aberrant telomere lengths might be key mechanisms in pediatric melanomas that need to be validated by large patient cohorts.

For PD-1 antibody combined with other standard chemotherapy regimens, no significant benefit was observed except for patient #10. Chemotherapy can potentially remodel the TME by depleting Tregs (26, 27), and increasing antigen presentation (28), however the precise mechanism is not well confirmed. There is some information for adult NK/T lymphoma treated with PD-1 antibody, but no data have been reported for NK/T lymphoma in patients under 18 years old, indicating that the present study provides a clinical reference. Kwong et al. used pembrolizumab in the treatment of 7 patients with relapsed and refractory NK/T-cell lymphoma, 5 patients achieved CR, and 2 patients achieved PR. The objective response rate (ORR) was 100%, which provided a new and effective salvage treatment scheme for relapsed and refractory patients (29).

Interestingly, among the patients treated with PD-1 antibody combined with decitabine, the effect was significant. The best efficacy was PR in 2 patients and CR in 1 patient. Epigenetic reprogramming can lead to impaired activation of anticancer immunity and protect tumor cells from immune surveillance (30). The hypomethylating agent decitabine is suggested to increase tumor T-cell infiltration and the antitumor response, ultimately restoring immunosurveillance. Nie et al. reported that decitabine plus camrelizumab (PD-1 antibody) treatment is safe and has a significantly higher CR rate in patients with relapsed/refractory cHL than camrelizumab alone (32% vs 71%, *p*=0.003) *(* 31). Moreover, another study conducted in pediatric and young adult patients with relapsed, refractory or progressive nonprimary CNS solid tumors and lymphomas is recruiting patients now. The study aimed to assess the safety and antitumor activity of the combination of pembrolizumab, decitabine and fixed-dose-hypofractionated index sited radiotherapy (NCT03445858) (32). Thus, combinatorial approaches are likely to be used in the future and have the potential to achieve therapeutic success (33, 34). At present, several clinical trials of anti-PD-1 combined with other immune checkpoint inhibitors in children’s tumors are also recruiting patients (NCT03668119, NCT 04416568, NCT 03837899 and NCT 03470922).

In this study, we found that treatment with PD-1 antibody alone was well tolerated in children. The patients lacked severe AEs compared to previously published literature, which may be due to racial differences. Yang et al. have found that the AEs of any grade with different prevalences between Asian populations and Western/International populations, which indicated that Asian populations that received PD-1 inhibitors have a lower rate incidence of fatigue, diarrhea and nausea and vomiting (35). Another reason that could explain the lack of severe AEs is the small number of patients who received PD-1 antibodies in the present study. In addition, some patients have a short course of treatment, and severe adverse effects might not be observed.

The different PD-1 antibodies and regimens patients received also induced toxicity profiles that differed from the published data. Wang et al. performed a systematic review and found that nivolumab appeared to have a higher mean incidence of all grade and grade 3 or higher adverse events than pembrolizumab (36). Li et al. compared the toxicity profiles between different PD-1 antibodies and found that some drug-specific AEs, such as reactive capillary hemangiomas for camrelizumab (58.6%), hyperglycemia for toripalimab (55.6%), and pembrolizumab had the highest incidence of grade 3–5 pneumonitis (37). However, the mechanism and clinical significance were unclear. Our study included patients who received sintilimab and toripalimab, both of which exhibited specific and different binding sites to human PD-1 compared to nivolumab and pembrolizumab (11, 38). The results of studies such as ORIENT-1 and CHECKMATE-205 indicated that sintilimab caused fewer toxicities in patients with r/r cHL than nivolumab and was proven safe (14, 39). However, comparing the safety of pembrolizumab and toripalimab in the POLARIS-01 and KEYNOTE-151 studies, it was observed that the toxicity profile was similar in melanoma (40, 41), with no novel adverse events identified. Thus, more studies are needed to drive confirm this conclusion in the future. However, we observed a greater frequency of grade 3 or grade 4 hematological toxicity in the cases treated with PD-1 antibody combined with chemotherapy, which we suspected was due to the side effects of chemotherapy regimens.

Our study had several limitations. First, it was a retrospective study with a small sample size. Patient selection bias may have impacted the outcomes reported here, as treatment decisions were likely influenced by previously published disease-specific PD-1 inhibitor response rates. Second, the pathological types of patients receiving PD-1 antibody combination therapy were diverse, making it difficult to compare the combination effects with PD-1 antibody alone. The combination effects may be the role of cytotoxic chemotherapy but not the PD-1 antibody that drove the response to therapy in any individual patient. Third, different patients used different antibodies against PD-1 for various reasons, which may lead to confounding factors in the analysis. Although PD-L1 expression and mutation load were not evaluated in this study, high levels of PD-L1 expression have been reported in adult (19, 42) and pediatric patients (43) with HL. Finally, because of the retrospective nature of the analysis, it was difficult to determine the causal relationship and level of adverse events based on the retrospective chart and to evaluate the safety profile. However, a phase I clinical study is being conducted by our institution and is underway for exploring the efficacy and safety of PD-1 antibody in patients with refractory or relapsed pediatric cancer (NCT04400851).

## Data Availability Statement 

The raw data supporting the conclusions of this article will be made available by the authors, without undue reservation.

## Ethics Statement 

The studies involving human participants were reviewed and approved by Ethics Committee of Sun Yat-sen University Cancer Center. Written informed consent to participate in this study was provided by the participants’ legal guardian/next of kin.

## Author Contributions 

YZ and YQ is responsible for the study design. YQ, JW, and JZ wrote the manuscript. HG and YQ is responsible for clinical data collection. JH, SL, FS, and LiaZ is responsible for the revision of the manuscript. LiZ participated in data analysis. ZZ and RC provided guidance in statistical analysis. XS and YZ reviewed the manuscript for intellectual content. All authors contributed to the article and approved the submitted version.

## Funding

This work was supported by the Key Technology Research Project of Guangzhou Science, Technology and Innovation Committee (No. 201902020001) and the Guangzhou Science and Technology project (No. 201905010004) and National Scientific Foundation of China (No. 82002835).

## Conflict of Interest

The authors declare that the research was conducted in the absence of any commercial or financial relationships that could be construed as a potential conflict of interest.
