# Supplementary material for: Combination Therapy With Anti-PD-1 or PD-1 Antibody Alone in Asian Pediatric Patients With Relapsed or Refractory Cancer
Source: Front Immunol. 2021 Jul 6;12:647733. doi: 10.3389/fimmu.2021.647733 (PMC8290852; doi:10.3389/fimmu.2021.647733)

**SUPPLEMENTARY MATERIAL**

**Supplementary Tables and figures**

**Supplementary Table 1. Patient Characteristics and response**

| **Patient ID** | **Age at diagnosis** | **Gender** | **KPS** | **Pathology** | **Stage at diagnosis** | **PD-1 antibody** | **Cycles**  **Of PD-1 monotherapy** | **Radiotherapy during treatment** | **Number of prior therapies** | **Best objective response** | **PFS**  **(days)** | **Survival**  **status** | **Pre-**  **radiotherapy** | **Pre-chemotherapy** |
| --- | --- | --- | --- | --- | --- | --- | --- | --- | --- | --- | --- | --- | --- | --- |
| 1 | 6 | M | 90 | Hodgkins lymphoma | III | Toripalimab | 7 | N | 2 | CR | 200 | alive | N | Y |
| 2 | 3 | M | 90 | Hodgkins lymphoma | III | Toripalimab | 7 | Y | 2 | PR | 316 | alive | N | Y |
| 3 | 7 | M | 90 | Hodgkins lymphoma | IV | Nivolumab | 16 | Y | 3 | SD | 628 | alive | Y | Y |
| 4 | 14 | F | 90 | Hodgkins lymphoma | IIB | Nivolumab | 8 | Y | 4 | PR | 1254 | alive | N | Y |
| 5 | 7 | M | 90 | Hodgkins lymphoma | IV | Nivolumab | 7 | N | 5 | CR | 1272 | alive | N | Y |
| 6 | 9 | M | 90 | Hodgkins lymphoma | IV | Nivolumab | 6 | N | 4 | CR | 1145 | alive | Y | Y |
| 7 | 5 | M | 80 | Melanoma | IV | Pembrolizumab | 4 | N | NA | SD | 301 | dead | N | N |
| 8 | 2 | M | 70 | Melanoma | III | Pembrolizumab | 2 | N | NA | PD | 62 | dead | N | N |
| 9 | 10 | M | 80 | Burkitt lymphoma | III | Nivolumab | 2 | N | 4 | PD | 34 | dead | N | Y |
| 10 | 11 | M | 90 | NK/T lymphoma | II | Toripalimab+PGMDE | 17 | Y | 1 | CR | 524 | alive | Y | Y |
| 11 | 3 | F | 70 | Atypicalteratoid／rhabdoid tumor，AT／RT | IV | Toripalimab+VII | 2 | N | 1 | SD | 90 | dead | N | Y |
| 12 | 10 | M | 90 | NK/T lmphoma | IV | Toripalimab+PGMDE | 2 | N | 1 | SD | 69 | dead | N | Y |
| 13 | 1 | M | 90 | Burkitt lymphoma | III | Nivolumab+Nab-paclitaxel+Liposome doxorubicin | 3 | N | 7 | PR | 37 | dead | Y | Y |
| 14 | 10 | M | 90 | Retroperitoneal neuroblastoma | IV | Sintilimab+Ifosfamide+Nedaplatin+Etoposide | 6 | N | 3 | SD | 106 | alive | Y | Y |
| 15 | 6 | M | 90 | Retroperitoneal rhabdomyosarcoma | IV | Toripalimab+Vincristine+Temozolomide+Irinotecan | 2 | Y | 4 | SD | 109 | dead | Y | Y |
| 16 | 6 | M | 90 | Burkitt lymphoma | III | Toripalimab+Nab-paclitaxel+Liposome doxorubicin | 1 | N | 2 | PD | 20 | dead | N | Y |
| 17 | 9 | F | 50 | Embryonal tumors of the central nervous system (CNS),NOS | IV | Toripalimab+Everolimus | 6 | N | 5 | PR | 90 | alive | Y | Y |
| 18 | 11 | M | 90 | Tongue cancer | IV | Toripalimab+carboplatin+5-fluorouracil | 8 | N | 5 | PD | 32 | dead | Y | Y |
| 19 | 15 | F | 90 | Embryonal rhabdomyosarcoma | Ⅲ | Toripalimab+Decitabine | 4 | N | 6 | CR | 362 | alive | Y | Y |
| 20 | 6 | M | 90 | Embryonal rhabdomyosarcoma | IV | Toripalimab+Decitabine | 4 | N | 8 | PR | 14 | dead | Y | Y |
| 21 | 8 | M | 90 | Germ cell tumors in the basal ganglia | IV | Pembrolizumab+vincristine+temozolomide+rinotecan | 5 | N | 5 | PD | 33 | dead | Y | Y |
| 22 | 12 | F | 90 | Lymphoepitheliomatoid-like carcinoma | IV | Toripalimab+Decitabine | 3 | N | 5 | PR | 448 | alive | N | Y |

**Supplementary Table 2. Adverse events - incidence and grading according to CTCAE v 4.03**

| Event* | Any grade  Number of patients(%） | Grade ≥3(PD-1 alone/ PD-1 combination) |
| --- | --- | --- |
| White blood cell decreased | 8/22(36.4%) | 0/3 |
| Neutrophil count decreased | 14/22(63.6%) | 0/3 |
| Anaemia | 8/22(36.4%) | 0/6 |
| Platelet count decreased | 3/22(13.6%) | 0 |
| Aspartate aminotransferase  increased | 2/22(9.1%) | 0 |
| Alanine aminotransferase  Increased | 2/22(9.1%) | 0 |
| Pyrexia | 5/22(22.7%) | 0/1 |
| Fatigue | 3/22(13.6%) | 0 |
| Hypothyroidism | 1/22(4.5%) | 0 |
| Hyperthyroidism | 2/22(9.1%) | 0 |
| Rash | 1/22(4.5%) | 0 |
| Headache | 2/22(9.1%) | 0 |

*The most common treatment-related adverse events included decreased white blood cell counts (36.4%, 8 patients), decreased neutrophil counts (63.6%, 16 patients), anemia (36.4%, 8 patients) and pyrexia (22.7%, 5 patients). No severe toxicity was observed in the patient cohort with anti-PD-1 treatment alone which indicated an acceptable safety profile. Among the severe TRAEs, most were hematologic toxicities with 3 patients having severe white blood cell decreases, 3 patients having severe neutrophil count decreases, 6 patients having severe anemia and 1 patient having severe pyrexia. One explanation for the serious side effects may be due to chemotherapy in the combination regimen.

**Supplementary Figure 1.** **Comparison of tumor burden of patient #10 before and after treatment.** Patient #10 had recurrent NK/T-cell lymphoma. Laryngoscopic examination revealed that white pseudomembranous neovascularization was found in the vocal cords, anterior commissures, posterior commissures and subglottic areas. The biopsy pathology results were consistent with NK/T lymphoma. PET/CT inspection indicated that the metabolism of mucosa above the glottic fissure was active and that the metabolism of multiple lymph nodes in bilateral regions II - III was slightly active. Bone marrow was normal. After PGMDE regimen chemotherapy combined with adjuvant radiotherapy (GTV4400cgy/20f and CTV3600cgy/20f were given), the curative effect was evaluated as CR, and tumor recurrence was reexamined after stopping treatment for 1.5 years. After 2 courses of treatment, the patient was evaluated as CR by MRI. And he achieved a metabolic of CR by PET. After 4 courses of treatment, patient #10 received nasal radiotherapy (GTV 4620/22F, ctv1 4180/22F, ctv2 3630/22F) followed by PD-1 antibody monotherapy. No tumor recurrence was found with imaging evaluation. The PFS time was 17 months.


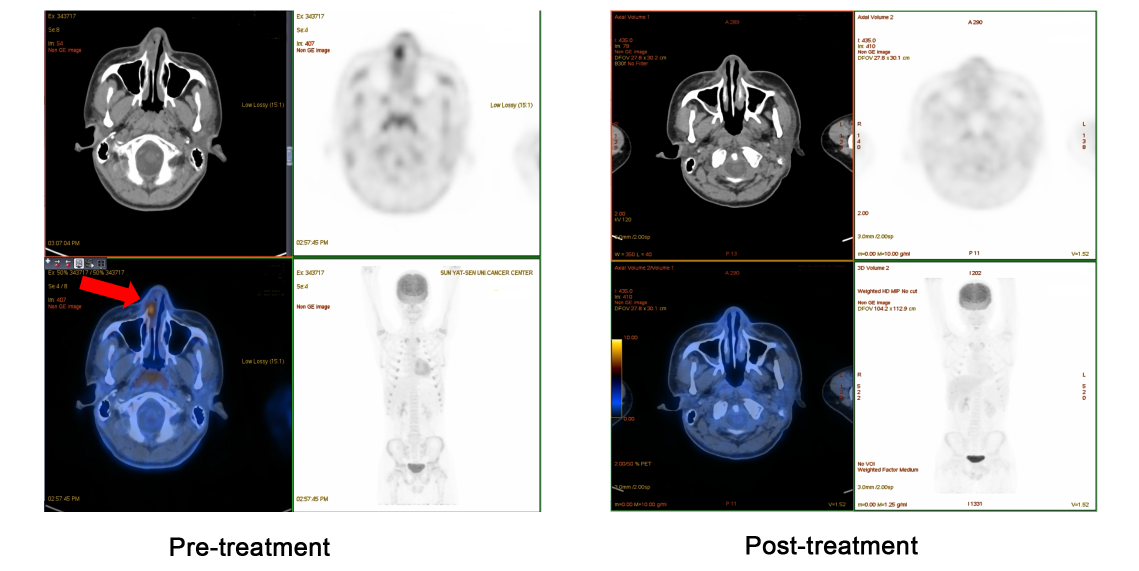


**Supplementary Figure 2. Response characteristics and changes in tumor burden in patients receiving PD-1 combination therapy.** A) Shown are best percentage changes from baseline in the sum of the longest diameter of target lesions in patients treated with PD-1 combination therapy with at least one post-baseline assessment. B) Swimmer’s Plot of time on treatment for 13 evaluable patients treated with PD-1 combination therapy.


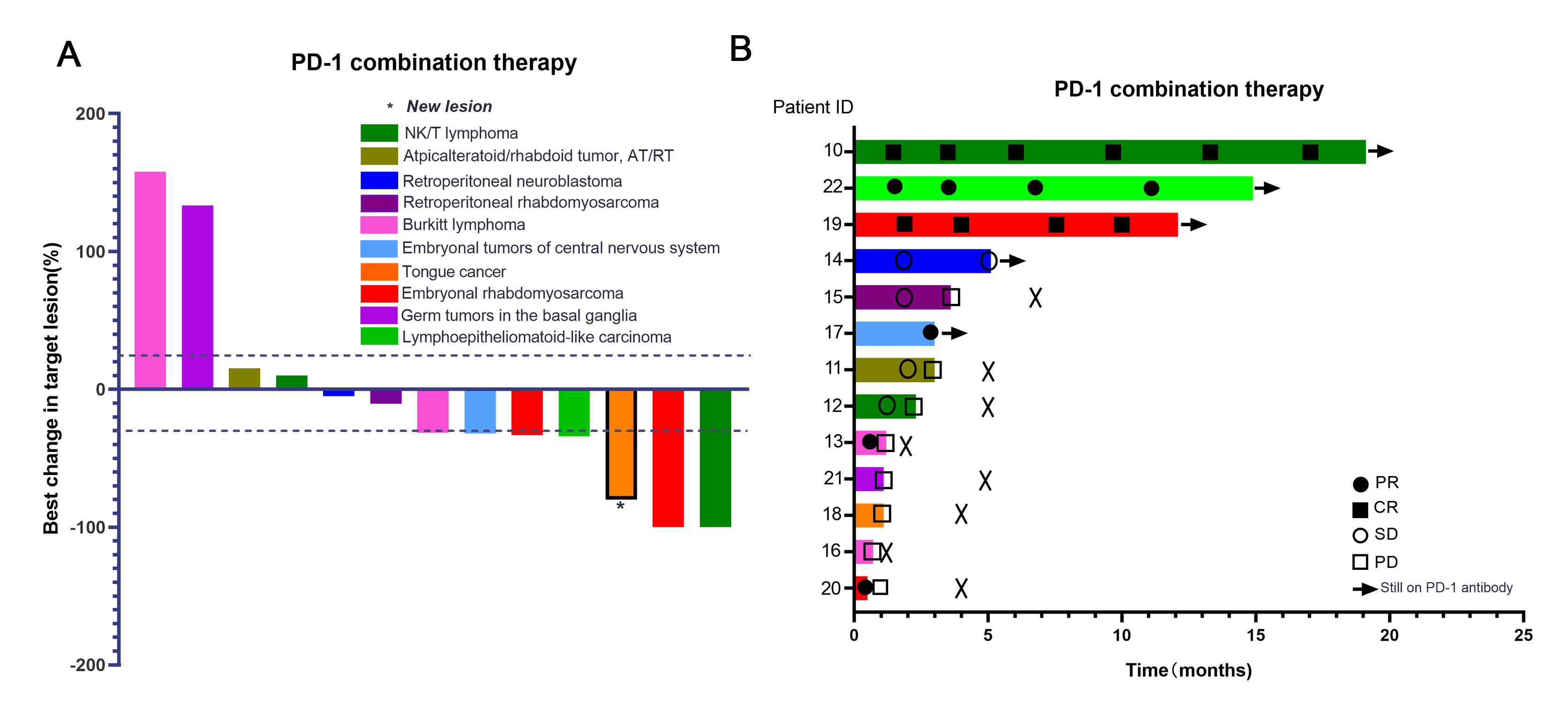

Supplement: Supplementary file 1 [file DataSheet_1.doc]
